# Quality of Supplementary Morning Lighting (SML) During Propagation Period Affects Physiology, Stomatal Characteristics, and Growth of Strawberry Plants

**DOI:** 10.3390/plants9050638

**Published:** 2020-05-16

**Authors:** Hao Wei, Chen Liu, Jiangtao Hu, Byoung Ryong Jeong

**Affiliations:** 1 Department of Horticulture, Division of Applied Life Science (BK21 Plus Program), Graduate School of Gyeongsang National University, Jinju 52828, Korea; oahiew@gmail.com (H.W.); chenliu215@gmail.com (C.L.); hujiangtao@gnu.ac.kr (J.H.); 2 Institute of Agriculture & Life Science, Gyeongsang National University, Jinju 52828, Korea; 3 Research Institute of Life Science, Gyeongsang National University, Jinju 52828, Korea

**Keywords:** chlorophyll, guard cell, light quality, LEDs

## Abstract

Artificial light supplementation is widely used in modern agriculture. Due to their numerous advantages, light emitting diodes (LEDs) are widely used to effectively increase the yield or control the development of crops. In the present study, the effects of supplementary morning lighting (SML) with LEDs on the physiology and stomatal characteristics of strawberry plants were studied, with the aim of awakening the plant guard cells before sunrise and enabling strawberry plants to efficiently photosynthesize immediately after sunrise. Young daughter plants of ‘Maehyang’ and ‘Seolhyang’ strawberry cultivars that have just rooted were grown under LEDs with different wavelengths—white (W), red (R), mixed blue and red (BR, 1:1), and blue (B)—to investigate the effects of the SML on the physiology, stomatal characteristics, and growth. The SML was provided for 2 h at an intensity of 100 μmol·m^−2^·s^−1^ PPFD before sunrise every morning. A group without supplementary lighting was set as the control. The results showed that the different SML qualities have significantly affected the stomatal characteristics. The B SML promoted the stomatal opening more effectively compared to the other SMLs. The stomatal conductance and quantum yield (Fv/Fm) of leaves treated with the SMLs were higher than those of the control group. The B and BR SMLs most significantly affected the stomatal conductance and quantum yield (Fv/Fm). After 30 days of the SML treatments, it was observed that the B SML effectively improved the plant quality, chlorophyll content, and carbohydrate accumulation in the two strawberry cultivars. In general, a short-term exposure to blue light before sunrise can effectively improve the quality and promote the production of strawberry plants.

## 1. Introduction

Strawberry, native to South America, was first bred in France and has since become a very valuable crop. It is now widely grown in the Republic of Korea, China, Europe, the United States, Japan, and other countries. The cultivation area and production of strawberry in the Republic of Korea are 5978 ha and 191,218 tons, respectively. The Republic of Korea has become the world’s sixth largest producer of strawberry [1]. In the Republic of Korea, strawberry was mainly cultivated in fields in the 1980s, which has since gradually transitioned to protected cultivation. By 2017, protected strawberry cultivation in the Republic of Korea accounted for 97.9% of the total cultivation area [2]. Gyeongnam is a major Korean strawberry-producer region that accounts for 44.5% (85,110 tons) of the country’s total production. Ninety-five percent of the strawberry production in the region is exported to other parts of the world, such as Southeast Asia. The strawberry cultivars that are mainly cultivated in Gyeongnam are ‘Maehyang’ and ‘Seolhyang’, which both have a high sugar content, easy dormancy breaking, and fast flower bud differentiation. Compared to ‘Seolhyang’, ‘Maehyang’ has harder fruits and thus is more suited for long-term storage and export. In Gyeongnam, strawberry is planted in September and fruits are harvested from November to May; daughter plants are cut in May or June and planted in September. The whole propagation period is three to four months, and methods with which to shorten the propagation time and improve the quality of daughter plants have become a valuable research topic for strawberries.

Artificial light has been widely used in horticulture, especially for greenhouse crops. At the present day, artificial light is generally applied in two forms. One form is to use artificial light as the sole light source to make plants grow. This is generally used in vertical crop cultivation, such as in plant factories, growth chambers, tissue culture rooms, etc. It has the advantage of being able to fully use the cultivation space. Another form is the use of artificial light sources to supplement light based on the natural light conditions, such as in underlit areas, winters with shorter photoperiods, rainy seasons with a lack of natural light, or to regulate the photoperiod of long-day or short-day plants. The aim of this form is to improve the crop yield, regulate flowering, etc. Nowadays, common sources of artificial lighting in horticultural applications are HPS (high pressure sodium lamp), MH (metal halid lamp), FL (fluorescent lamp), and LED (light emitting diode). LEDs have the advantages of a high wavelength controllability, relatively low heat release, low power consumption, long use time, and flexible size design. LEDs are increasingly replacing other light sources due to their advantages. Wojciechowska et al. compared several commonly used artificial light sources in terms of yield and energy consumption and found that mixed LEDs were an effective way to improve production efficiency of *Valerianella locusta* L. [3]. The authors also have previously investigated the effects of the source, intensity and duration of light on the growth and development of crops, and have come to the conclusion that LEDs are superior to other light sources during the seedling production period of vegetables crops [4,5,6].

White light, red light, blue light, green light, infrared light, and ultraviolet light have different wavelengths and different impacts on the plant growth and development. Among these lights, red and blue lights are the main spectral range for plant photosynthesis and have the most significant impacts. Red light can greatly enhance photosynthesis and be conducive to plant growth, but too much of it may cause excessive growth of stems and/or branches, and blue light can promote the activity of chloroplasts and enhance photosynthesis [7]. Moreover, blue light is known to be very important for plant differentiation and stomatal regulation. A stomate consists of two guard cells that form a hole which opens and closes, and most stomata are located on the back of plant leaves [8]. Usually light, temperature, CO_2_ concentration, water deficit, and abscisic acid (ABA) influence the stomatal movement [9]. Stomata are the main channels of gas exchange between plant leaves and their environment. Gases such as CO_2_, O_2_, and water vapor diffuse through pores [10]. Water evaporation through the stomata creates motive power that allows the water absorbed by the roots to be transported to leaves, thus transporting nutrients to the entire plant. In a word, stomata play a key role in controlling the balance between water loss and the biomass production [11].

In nature, the air temperature is at its lowest before sunrise each day. At this time, almost all stomata of plants are closed in the low temperature and dark environment. The light intensity reaches the light compensation point of photosynthesis after sunrise, but the air temperature rises more slowly than the light intensity does. Thus, most stomata remain closed until the temperature rises, preventing CO_2_ from entering the leaves, limiting the ability of plants to make full use of the available sunlight for carbon fixation. Since different light qualities have significant effects on the photosynthesis and stomatal movement in plants, the authors propose to use a short-term supplementary light before sunrise to awaken plants in advance and help plants make full use of the sunlight to carry out photosynthesis after sunrise, thereby increasing the crop yield or shortening the production cycle. In addition, Hanyu and Shoji [12] have found that supplemental lighting at the beginning and the end of the dark period can promote the growth of spinach. This suggests that the light of BOD (beginning-of-day) or EOD (end-of-day) effect is present among plants. Therefore, the effects of the light supplement before sunrise during the propagation period on the photosynthesis, stomatal movement, growth, and development of two strawberry cultivars ‘Maehyang‘ and ‘Seolhyang‘ were investigated. The aim of this study is to find out the best light source to awaken plants in advance and improve the photosynthetic efficiency of strawberry plants during the propagation stage.

## 2. Materials and Methods

### 2.1. Plant Materials, Light Treatments, and Culture Conditions

**Figure 1:**
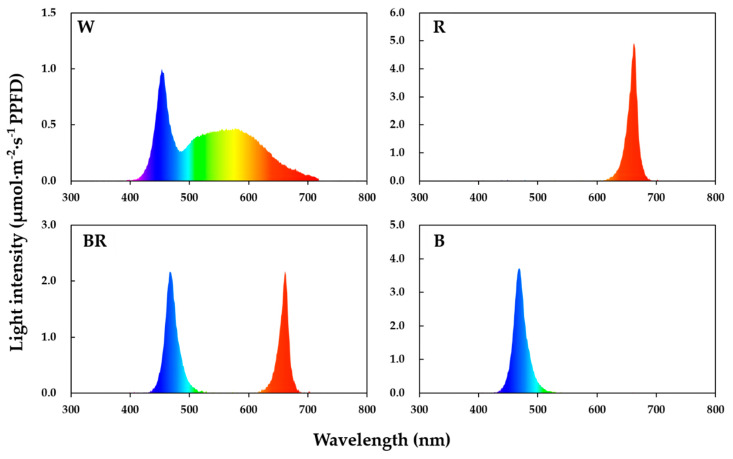
Spectral qualities of the different supplementary morning lighting (SML) sources used in the experiment. W (white LEDs), R (red LEDs), BR (mixed blue and red LEDs), and B (blue LEDs), measured with a portable spectroradiometer (Spectra Light ILT 950, International Light Technologies, Inc., Peabody, MA, USA).

Two strawberry cultivars ‘Maehyang’ and ‘Seolhyang’, which are widely cultivated in the Republic of Korea, were used in this experiment. Runners of similar sizes were separated from the mother plants and planted in a 21-cell tray filled with the BVB (Bas Van Buuren Substrates, De Lier, The Netherlands) substrate, then put into a fogging tunnel to root. Ten days later, well-rooted daughter plants were taken out of the fogging tunnel and transferred to a seedling bench with LEDs in a venlo type glasshouse. Light with an intensity of 100 μmol·m^−2^·s^−1^ PPFD from white (W), red (R), mixed blue and red (BR, 1:1), and blue (B) LEDs (custom made, SungKwang LED Co., Ltd., Incheon, Republic of Korea) was provided as the supplementary light. A group without any supplementary light was set as the control. There was enough space between the benches to ensure that no spectrum from other treatments can be detected in each treatment bench, thus the treatments did not interfere with each other. Light intensity was measured with a quantum radiation probe (FLA 623 PS, ALMEMO, Holzkirchen, Germany) at the top-leaf-level of the plants. The spectral distributions (Figure 1) of the LEDs were measured with a portable spectroradiometer (Spectra Light ILT 950, International Light Technologies, Inc., Peabody, MA, USA). A timing controller (SJP-CP16H, Seojun Ltd., Seoul, Republic of Korea) was used to control the entire circuit and to make sure that the LEDs were lit for 2 h before sunrise every morning. The experiment lasted for one month and the plants were fed with a greenhouse multipurpose nutrient solution daily. The cultivation environment had 25/15 °C day/night temperatures, 70% relative humidity, and a natural photoperiod of 12 h.

### 2.2. Measurements of the Growth Parameters

After 30 days of the SML treatments, the growth parameters such as length, fresh and dry weights, crown diameter, number of leaves and runners, length, fresh and dry weight of roots were measured. The shoot length was measured as the length from the crown to the top of the plant, and the root length was measured as the length from the crown to the bottom of the roots. Both fresh and dry (dried in an oven at 70 °C for 72 h) weights were measured with an electronic balance (ENTRIS224I-1S, Sartorius, Goettingen, Germany). The crown diameter was measured with a digital Vernier caliper (CD-20CPX, Mitutoyo, Kawasaki, Japan). A fully unfolded leaf was considered as one leaf.

### 2.3. Scanning Electron Microscopy (SEM) of Stomata

After 2 h of the SML treatments, the second fully expanded new healthy leaf close to the same position near the main vein was punched out into pieces with a 7.8 mm diameter, and immediately fixed in 2.5% (*v*/*v*) glutaraldehyde (pH 7.5) for 8 h in a 4 °C refrigerator. The samples were washed three times with a 0.1 M PBS (phosphate-buffered saline), and then post fixed in 1.0% (*w*/*v*) osmium tetroxide (pH 7.2) for 2 h at 4 °C. The samples were subsequently washed with the PBS again and dehydrated in order with 30%, 50%, 70%, and 90% ethanol solutions in 20-min intervals, then with 100% ethanol for another 8 h. Finally, the samples were washed with acetone and dried naturally at room temperature. Leaf fragments with the back side on were positioned on stubs prior to gold coating in a sputter coater (SC 7640, Polaron, Sussex, UK). A sputtering current of 20 mA was applied for 4 min, giving a gold coating with a thickness of approximately 22 nm. Samples were observed and photographed with a scanning electron microscope (JSM-6380LV, JEOL, Tokyo, Japan) at 15 kV. The epidermis including the stomata in the leaves were observed at a 2000× magnification.

### 2.4. Stomatal Conductance and Quantum Yield (Fv/Fm)

The stomatal conductance was assessed after 2 h of the SML treatments using a Decagon Leaf Porometer SC-1 (Decagon Device Inc., Pullman, WA, USA). The quantum yield was determined after 2 h of the SML treatments with a FluorPen FP 100 (Photon Systems Instruments, PSI, Drásov, Czech Republic).

### 2.5. Soluble Sugars, Starch, and Soluble Proteins

The anthrone colorimetric method was used for the determination of contents of the soluble sugars and starch extracts [13]. The 0.2 g of fresh leaf samples were weighed and ground, then added with 20 mL of distilled water, and extracted at 100 °C for 30 min. After 15 min of centrifugation at 6500 rpm, the solution was filtered, then the filtrate was brought up to 100 mL with distilled water in a 100 mL volumetric flask for the measurement of soluble sugars. After that, 20 mL of a distilled water insoluble substance was added and re-extracted for 15 min at 100 °C, then mixed with 2 mL of perchloric acid (52% *v*/*v*) for another 15 min at 100 °C. The solution was subsequently filtered, and the volume of the solution was brought up to 100 mL with distilled water in a 100 mL volumetric flask for the starch measurement.

For the measurement of the soluble sugars, 0.2 mL of the extracting solution was mixed with 1.8 mL of distilled water, 0.5 mL of anthrone (2% *v*/*v*), and 5.0 mL of sulfuric acid (98% *v*/*v*). After 10-min of incubation at 100 °C, the absorbance was recorded with a UV-spectrophotometer (Libra S22, Biochrom Ltd., Cambridge, UK) at 630 nm.

For the measurement of the starch, 1.0 mL of the starch solution was mixed with 1.0 mL of distilled water, 1.0 mL of anthrone, and 5 mL of sulfuric acid (98% *v*/*v*). After the solution was incubated at 100 °C for 10 min, the absorbance was recorded with a UV-spectrophotometer at 430 nm. The calibration curves of soluble sugars and starch were made by their respective standard solutions.

To further compare the metabolism of strawberry in different treatments, the total soluble proteins were measured by the method of Bradford [14]. The 0.1 g of fresh leaf samples were ground with liquid nitrogen. The leaf powder was extracted for 10 min with a 1.5 mL sodium phosphate buffer. The homogenate was centrifuged at 13,000 rpm for 20 min at 4 °C. After centrifugation, the supernatant was transferred to a new tube with a pipette for the next step of assays. For protein estimation, 50 µL of the supernatant was mixed with 1450 µL of the Braford reagent and incubated at room temperature for 10 min. The absorbance was recorded with a UV-spectrophotometer at 595 nm. The calibration curve of proteins was made by a bovine serum albumin (BSA) as the standard.

### 2.6. Measurements of Chlorophyll A and B Contents

The chlorophyll a and b contents were estimated according to the method of Arnon [15]. The 1 g fresh leaf samples were cut into small pieces and homogenized in a precooled mortar and pestle using 80% (*v*/*v*) acetone. The extract was centrifuged at 3000 rpm for 15 min and the volume of the solution was brought up to 25 mL with 80% (*v*/*v*) acetone. The supernatant was then transferred to a colorimeter tube and the absorbance was measured at 645 and 663 nm, against an 80% (*v*/*v*) acetone blank in a UV-spectrophotometer. The chlorophyll a and b levels were determined using the following equations.

Chlorophyll a = (12.7 × OD at 663 nm) − (2.69 × OD at 645 nm)(1)

Chlorophyll b = (22.9 × OD at 645 nm) − (4.08 × OD at 663 nm)(2)

The chlorophyll content was expressed as mg of chlorophyll per g of fresh leaf weight.

### 2.7. Data Collection

After the parameters were measured, the Dickson quality index (DQI) was also calculated, as shown below [16,17,18,19]:

(3) $$Dickson\;quality\;index\;\left(DQI\right)=\frac{Total\;plant\;dry\;weight\;\left(g\right)}{\frac{Height\;\left(mm\right)}{Crown\;diameter\;\left(mm\right)}+\frac{Shoot\;dry\;weight\left(g\right)}{Root\;dry\;weight\;\left(g\right)}}$$

The experiment was laid out in a completely randomized design with three replications per treatment, each of which consisted of 21 plants per plug tray. The treatment locations in a controlled environment were randomly laid out to minimize the location effects. Significant differences among the treatments were determined by analysis of variance (ANOVA) followed by the Tukey’s multiple range test at a significance level of *p* = 0.05 with the Statistical Analysis System (SAS, V. 9.1, Cary, NC, USA).

## 3. Results

### 3.1. Stomatal Morphology and Characteristics

**Figure 2:**
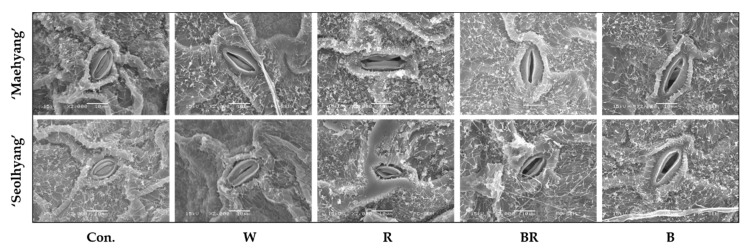
The effects of the SML quality on the stomatal morphology in new leaves of strawberry ‘Maehyang’ and ‘Seolhyang’ observed with a scanning electron microscope. W, R, BR, and B respectively represent white, red, mixed blue and red (1:1), and blue LEDs, and Con. indicates the control group without any SML. The samples were observed and photographed with a scanning electron microscope at 15 kV. The epidermis, including the stomata in leaves, was observed at a 2000× magnification.

To investigate the effects of the SML quality on the stomatal movement and characteristics, an ultrastructural tissue analysis was conducted with scanning electron microscopy (SEM) (Figure 2). The results showed that the stomata of the control group were almost completely closed, whereas the stomata of plants treated with the different SMLs were open. An analysis of the stomatal size of plants treated with the different SMLs are shown in Table 1. For ‘Maehyang’, the stomatal size was the largest when treated with the B SML, followed in order by the BR, R, and W SMLs. For ‘Seolhyang’, there was no significant difference in the stomatal size when treated with the B and BR SMLs, the values of which were higher than those of plants treated with the R and W SMLs. The length and width measurements of the guard cells showed that the SML had no significant effects on the width but affected the length of the guard cells. The guard cells treated with the SMLs in ‘Maehyang’ were significantly longer than those of the control group. The guard cells of ‘Seolhyang’ treated with the R and B SMLs were the longest. The effects of the SMLs on the stomatal density were statistically analyzed. For ‘Maehyang’, the stomatal density was the highest when treated with the B SML, not significantly different when treated with the W and BR SMLs and was the lowest when treated with the R SML. For ‘Seolhyang’, plants treated with the R, BR, and B SMLs had relatively high stomatal densities, compared to the control group and plants treated with the W SML.

### 3.2. Stomatal Conductance and Quantum Yield (Fv/Fm)

**Figure 3:**
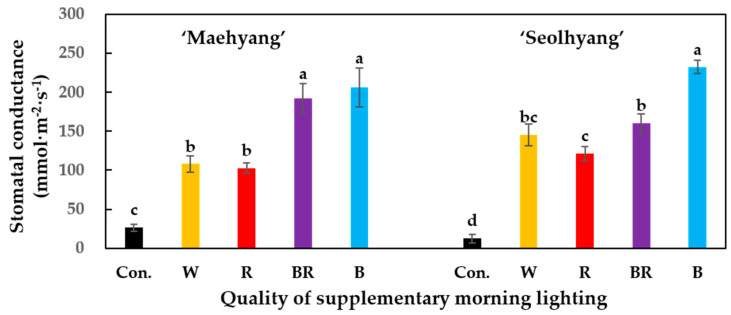
The effects of the SML quality on the stomatal conductance of the two strawberry cultivars. W, R, BR, and B respectively represent white, red, mixed blue and red (1:1), and blue LEDs, and Con. indicates the control group without any SML. Vertical bars are the means ± SE (*n* = 3). Mean separation within columns are significantly different according to Tukey’s multiple range test at *p* = 0.05.

**Figure 4:**
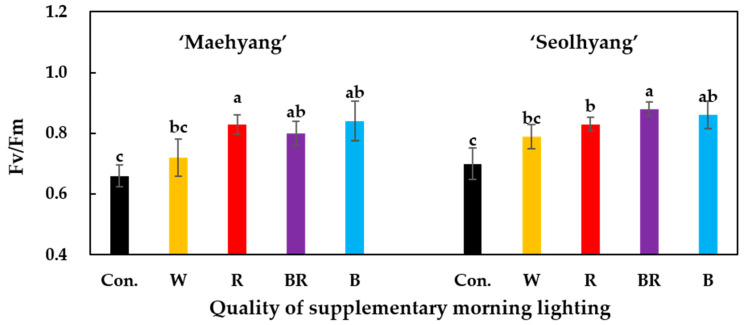
The effects of the SML quality on the quantum yield (Fv/Fm) of the two strawberry cultivars. W, R, BR, and B respectively represent white, red, mixed blue and red (1:1), and blue LEDs, and Con. indicates the control group without any SML. Vertical bars are the means ± SE (*n* = 3). Mean separation within columns are significantly different according to Tukey’s multiple range test at *p* = 0.05.

After the SML treatments, the stomatal conductance of strawberry leaves was measured (Figure 3). For both strawberry cultivars, the B SML led to a significantly higher stomatal conductance. There was no significant difference between the stomatal conductance of ‘Maehyang’ plants treated with the BR and B SMLs. The W and R SMLs led to relatively lower stomatal conductance values compared to the BR and B SMLs, but all SML treatments resulted in significantly higher stomatal conductance values than that of the control group. By measuring the chlorophyll fluorescence ability after the SML treatments (Figure 4), it was found that ‘Maehyang’ treated with the R, BR, and B SMLs had no significant difference in the quantum yield (Fv/Fm), the values of which were significantly higher than that of plants treated with the W SML and of the control group. For ‘Seolhyang’, the BR and B SMLs resulted in no significant difference in the quantum yield, the values of which were significantly higher than those of plants treated with the R and W SMLs. ‘Seolhyang’ treated with the R SML had a significantly higher quantum yield than the control group.

### 3.3. Growth and Development Parameters

The growth and development parameters (Table 2) of the two strawberry cultivars were measured after 30 days of the SML treatments. The shoot length of the control group was significantly higher than those of the SML-treated groups. For ‘Maehyang’, the B SML resulted in the lowest shoot length. For ‘Seolhyang’, the different SMLs did not lead to significant differences in the shoot length. For both cultivars, the B SML yielded the greatest shoot fresh weight, and the control group had the lowest shoot fresh weight. The fresh weights of ‘Seolhyang’ treated with the W and R SMLs were similar, which were lower than that of plants treated with the BR SML. For ‘Maehyang’, the shoot dry weight was lower for the control group, and those treated with the B and BR SMLs had the higher shoot dry weight. ‘Seolhyang’ plants treated with the B SML had the greatest shoot dry weight, followed by those treated with the BR SML. ‘Seolhyang’ plants treated with the W and R SMLs had the third greater shoot dry weights, the values of which did not significantly differ. Plants treated with the B SML also had the greatest crown diameter. The number of leaves for the two cultivars was not significantly affected by the SML quality, and the control group for ‘Maehyang’ had the least leaves. For both cultivars, all SML treatments resulted in longer roots compared to those of the control groups, where the B and BR SMLs were the most effective in increasing the root length. The B and BR SMLs resulted in the highest root fresh and dry weights for ‘Maehyang’, while the B SML led to a significantly higher root fresh and dry weights compared to plants in all other treatments for ‘Seolhyang’. In addition, different SML qualities also had a certain impact on the development of strawberry runners. For ‘Maehyang’, plants treated with the B, BR, and R SMLs had more runners than those treated with the W SML and the control group. For ‘Seolhyang’, the B and BR SMLs resulted in the highest number of runners, while the control group had the lowest number of runners. The number of runners of ‘Seolhyang’ plants treated with the R and W SMLs did not significantly differ.

**Figure 5:**
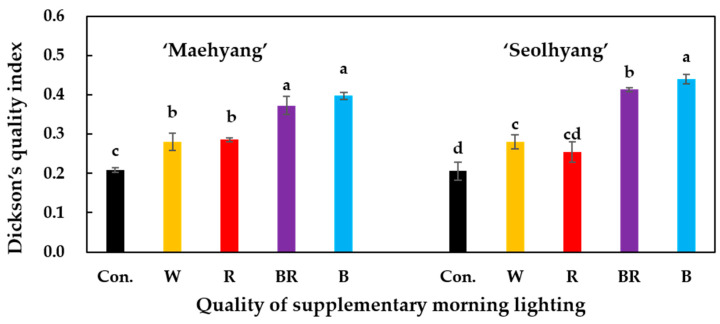
The effects of the SML quality on the Dickson quality index (DQI) of the two strawberry cultivars. W, R, BR, and B respectively represent white, red, mixed blue and red (1:1), and blue LEDs, and Con. indicates the control group without any SML. Vertical bars are the means ± SE (*n* = 3). Mean separation within columns are significantly different according to Tukey’s multiple range test at *p* = 0.05.

The DQI of the two strawberry cultivars after the 30-day SML treatments was calculated with the growth and development parameters presented above (Figure 5). It was found that for ‘Maehyang’, the DQI was significantly higher for plants treated with the B and BR SMLs, followed in order by those treated with the W and R SMLs, and the control group. For ‘Seolhyang’, plants treated with the B SML had the highest DQI, followed in order by those treated with the BR, W, R SMLs, and the control group.

### 3.4. Chlorophyll Contents

**Figure 6:**
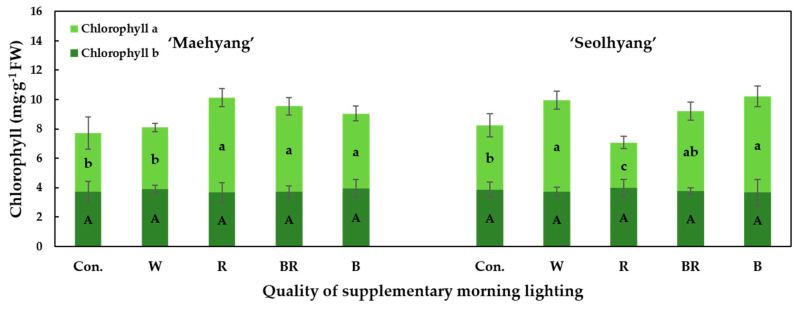
The effects of the SML quality on the chlorophyll a and b contents in leaves of strawberry ‘Maehyang’ and ‘Seolhyang’. W, R, BR, and B respectively represent white, red, mixed blue and red (1:1), and blue LEDs, and Con. indicates the control group without any SML. Vertical bars are the means ± SE (*n* = 3). Mean separation within columns are significantly different according to Tukey’s multiple range test at *p* = 0.05.

The chlorophyll a and b contents in the leaves of strawberry plants treated with the different SMLs were analyzed (Figure 6). In general, the SML did not significantly affect the chlorophyll b content in the two strawberry cultivars. For ‘Maehyang’, the R, BR, and B SMLs significantly increased the chlorophyll a content, while for ‘Seolhyang’, the W, B, and BR SMLs led to relatively high chlorophyll a contents, and the R SML resulted in the lowest chlorophyll a content.

### 3.5. Contents of the Soluble Sugars, Starch, and Soluble Proteins

**Figure 7:**
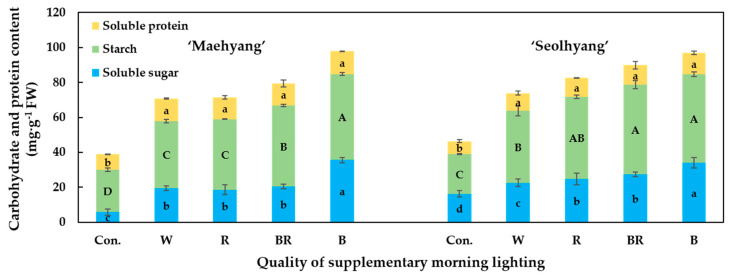
The effects of the SML quality on the contents of the soluble sugars, starch, and soluble proteins in the leaves of strawberry ‘Maehyang’ and ‘Seolhyang’. W, R, BR, and B respectively represent white, red, mixed blue and red (1:1), and blue LEDs, and Con. indicates the control group without any SML. Vertical bars are the means ± SE (*n* = 3). Mean separation within columns are significantly different according to Tukey’s multiple range test at *p* = 0.05.

The contents of the soluble sugars, starch, and soluble proteins affected by the SML are presented in Figure 7. It was found that the SML treatments increased the contents of the soluble sugars, soluble proteins, and starch. For ‘Maehyang’, the contents of soluble sugars and starch were the highest in plants treated with the B SML. There was no significant difference in the contents of soluble sugars in plants treated with the W, and R and BR SMLs, but the starch content in plants treated with the BR SML was higher than those in plants treated with the W and R SMLs. For ‘Seolhyang’, plants treated with the B SML had the highest soluble sugar contents. There was no significant difference in the soluble sugar contents in plants treated with the R and BR SMLs, the values of which were higher than those of plants treated with the W and B SMLs. There was no significant difference in the starch contents in plants treated with the R and BR SMLs, and the starch contents in plants treated with the BR and B SMLs were higher than that of plants treated with the W SML. Except for the control group, there was no significant difference in the contents of soluble proteins in strawberry plants treated with the SMLs.

## 4. Discussion

The light quality significantly impacts the growth and development of plants. In horticultural production, growers have been widely using LEDs to supplement different qualities of light for crops according to the production demands, such as increasing the crop yield, regulating the flowering period, or inhibiting overgrowth, etc. Plants recognize light changes by using signal-transducing photoreceptors including phytochromes, cryptochromes, and phototropins [20]. Responses of photoreceptors evoked by the spectral composition directly or indirectly affect the physiological, morphological, and anatomical features of plants. Each stomate, surrounded by a pair of guard cells, regulate the CO_2_ uptake and water loss from leaves [21,22,23]. Stomatal opening is driven by the accumulation of K^+^ salts and sugars in the guard cells, which is mediated by the electrogenic proton pumps in the plasma membrane and/or the metabolic activity [24,25,26]. Opening responses are achieved by the coordination of light signaling, light-energy conversion, membrane ion transport, and metabolic activity in guard cells. In leaves, the stomata typically open during the day to favor CO_2_ diffusion when light is available for photosynthesis, and close at night to limit transpiration and save water [27]. 

In this study, we observed the stomatal morphology, stomatal density, and stomatal conductance, and found that the stomata of strawberry leaves in the control group were almost closed before sunrise, which was mainly caused by darkness and low temperature. Stomatal closure induced by darkness is inhibited by the H_2_O_2_-scavenging enzyme catalase or the antioxidant N-acetyl cysteine, or by diphenylene iodonium, an inhibitor of the H_2_O_2_-generating enzyme NADPH oxidase [28]. Wilkinson et al. [29] found that the cold-induced stomatal closure exhibited by intact *Commelina communis* leaves increases the apoplastic calcium uptake by guard cells. Zeiger and Hepler [30] found that onion guard cell protoplasts swell when illuminated with blue light. The response is a 35% to 60% increase in the volume and is dependent on K^+^. Epidermal cell protoplasts do not swell under the same conditions. It is postulated that a membrane-bound blue photoreceptor mediates a direct response of guard cells to light. Takemiya et al. [31] reported that phototropins—blue light receptors in plants—mediate the stomatal opening through the activation of the plasma membrane H+-ATPase in *Vicia faba*. In 1997, the blue-light receptors LOV1 and LOV2 for phototropism were identified by the Briggs group [32] and were subsequently renamed phototropins 1 (phot1) and 2 (phot2), respectively [33]. In guard cells, phot1 and phot2 contribute to the blue light-induced stomatal opening when irradiated at fluence rates higher than 1 μmol·m^−2^·s^−1^ PPFD [34]. On the other hand, the stomata of strawberry leaves treated with the SMLs for 2 h had different degrees of stomatal opening, among which the stomata of leaves treated with the B SML had the highest degree of opening. This suggests that the spectra of several tested visible lights have the ability to induce stomatal opening, and blue light is the most effective in inducing stomatal opening. The quantum yield (Fv/Fm), which can reflect the chlorophyll fluorescence ability after the SML treatments, showed that the B SML also promoted the chlorophyll fluorescence ability of both strawberry cultivars, which indirectly reflected the enhancement of the photosynthetic ability.

Shimazaki et al. [35] described the mechanisms with which light regulates the stomatal movement and mentioned that blue light enhances stomatal CO_2_ fixation under sun flecks in the canopy [36]. Moreover, a short exposure to blue light will enhance the light-energy capture by providing CO_2_ when the leaf encounters successive sun flecks [37]. Red light also promotes the stomatal opening in plants. In the orchid *Paphiopedilum*, a low photon flux density of red light promotes the stomatal opening [38]. However, the stomatal opening response requires a high intensity red light in most plant species [39]. However, it has been found in numerous species that stomatal conductance in leaves under a combination of red and blue lights is typically larger than the sum of the stomatal conductance under blue and red lights alone, suggesting a synergistic action of the stomatal opening in intact leaves [40,41,42,43,44,45]. In this study, the BR SML did not seem to affect the stomatal opening as much as the B SML did. Based on Shimazaki’s description [35], it is suspected that this is due to the intensity of supplementary light. Red light needs a higher intensity than blue light to promote stomatal opening. With the BR SML in this study, the intensity of the blue and red lights was 1:1, at 50 μmol m^−2^ s^−1^ PPFD. Compared with the 100 μmol·m^−2^·s^−1^ PPFD of the B SML, the BR SML was relatively less effective in promoting the stomatal opening. White light is a combination of lights of different wavelengths. It affects plants in a more complex way than blue and red lights do. According to the authors’ inferences, white light has relatively milder effects on the stomata compared to the other monochromatic lights because certain parts of the white light’s spectrum has an inhibitory effect on the stomatal opening. For example, Frechilla et al. [46] found that green light suppresses the stomatal opening in the epidermal strips of *Arabidopsis* under blue light. In summary, the B SML is the most effective in promoting the stomatal opening of strawberry leaves before sunrise, so that strawberry plants can absorb sufficient CO_2_ and photosynthesize immediately after sunrise.

In general, the 30-day SML treatments resulted in significantly improved growth and development parameters, quality, and carbohydrate accumulation in the two strawberry cultivars. The B SML was generally more effective in improving the plant qualities mentioned above, compared to the other SMLs. Previous studies have found that the light quality influences the growth of cells and tissue, photosynthetic characteristics, [47] crop yield, physiological and morphological qualities, stress regulation, and leaf aging [48]. The quality and quantity of light have been shown to alter the structure and function of chloroplasts in leaves [49,50,51]. This is reflected in the results of this study, especially in the case of ‘Maehyang’, where the B and BR SMLs led to a lower plant height and higher biomass. Based on these growth parameters, the DQI, which reflects the plant quality, was calculated. The plant quality was significantly higher when treated with the B SML than with the other SMLs. This indicates that a short-term exposure to blue light before sunrise promotes the growth and development of strawberry plants. Previous studies have also suggested the role of blue light in reducing cell expansion, and inhibiting leaf growth and stem elongation [52]. Mortensen and Stromme [53] found that the height of tomato plants significantly decreased under blue light enriched conditions. 

Hanyu and Shoji [12] determined that supplementary blue light at the end of the dark period results in a 20% increase of the total dry matter in spinach. Sung and Takano [54] and Sung et al. [55,56] found that a 5-min exposure to 30 μmol·m^−2^·s^−1^ PPFD blue light causes the greatest growth acceleration. It was also found that strawberry plants treated with the B SML had the highest total contents of soluble sugars, starch, and soluble proteins. In addition, Lopez-Juez and Hughes [57] showed that the chlorophyll a/b ratio in pea seedlings increases under an increased blue light, suggesting that the light acclimation responses are controlled by the blue light photoreceptors. In this study, it was also found that the different SML treatments affected the chlorophyll a content but did not significantly affect the chlorophyll b content in strawberry leaves. For both strawberry cultivars, the B SML led to a significantly higher chlorophyll a content compared to the other SMLs. With the exception of ‘Seolhyang’ treated with the R SML, the different SMLs increased the chlorophyll a content in the leaves of both strawberry cultivars.

Plants may recognize the onset of the light period by sensing blue light, as the blue fraction of the natural photosynthetic photon flux is the highest at sunrise [58]. Blue light enhances the transcription levels of *rbcs*, *rubisco*, and *cytochrome f*, and promotes the stomatal opening [57,59]. Moreover, a short-term supplement of blue light apparently optimizes the photosynthetic activity and consequently increases the photosynthetic efficiency during the light period [53]. This study verified that a short-term supplement of blue light before sunrise activated photosynthesis such that plants could immediately, and efficiently use the natural light for CO_2_ fixation after sunrise. This can help improve the quality and yield of strawberry plants during the propagation stage.

## 5. Conclusions

It can be seen that in the early growth stage of strawberry plants, a 2-h supplementary morning lighting with blue light at an intensity of 100 μmol·m^−2^·s^−1^ PPFD before sunrise helps strawberry plants produce biomass and improves the quality of plants, by promoting the stomatal opening and accumulation of the photosynthetic products.

## Figures and Tables

**Table 1:** The effects of the SML quality on the stomatal characteristics of strawberry ‘Maehyang’ and ‘Seolhyang’. W, R, BR, and B respectively represent white, red, mixed blue and red (1:1), and blue LEDs, and Con. indicates the control group without any SML. Mean separation within columns are significantly different according to Tukey’s multiple range test at *p* = 0.05.

Cultivar	Light Quality	Stomatal Density (mm^−2^)		Guard Cell Size (μm)				Stomatal Pore Size (μm)	
				Length		Width			
‘Maehyang’	Con.	194.3	b	12.3	b	2.4	a	0.0	e
	W	186.2	b	13.7	a	2.6	a	0.2	d
	R	160.6	c	14.2	a	2.4	a	0.8	c
	BR	184.0	b	14.3	a	2.9	a	2.0	b
	B	226.5	a	13.7	a	3.0	a	3.3	a
‘Seolhyang’	Con.	190.5	b	13.0	b	3.1	a	0.0	d
	W	180.0	b	13.6	b	2.9	a	0.6	c
	R	210.0	ab	14.6	a	2.9	a	1.2	b
	BR	236.7	a	12.5	c	2.6	a	3.1	a
	B	200.2	ab	15.4	a	3.2	a	3.4	a

**Table 2:** The effects of the SML quality on the growth and development parameters of strawberry ‘Maehyang’ and ‘Seolhyang’. W, R, BR, and B respectively represent white, red, mixed blue and red (1:1), and blue LEDs, and Con. indicates the control group without any SML. Mean separation within columns are significantly different according to Tukey’s multiple range test at *p* = 0.05.

Cultivar	Light Quality	Shoot Length (cm)		Shoot Fresh Weight (g)		Shoot Dry Weigh (g)		Crown Diameter (mm)		Leaf No.		Root Length (cm)		Root Fresh Weight (g)		Root Dry Weight (g)		Runner No.	
‘Maehyang’	Con.	32.3	a	15.20	d	1.08	c	2.56	d	8	b	20.6	c	24.60	c	1.68	c	2	b
	W	28.3	b	18.52	bc	1.22	b	2.86	c	10	a	22.6	b	32.31	b	1.75	b	2	b
	R	26.9	b	16.52	c	1.16	b	2.94	b	9	a	19.9	c	30.60	b	1.66	c	3	a
	BR	26.4	bc	19.36	b	1.45	a	3.15	ab	10	a	23.5	ab	36.62	a	1.95	a	3	a
	B	24.6	c	20.46	a	1.48	a	3.26	a	10	a	24.8	a	35.80	a	1.84	ab	3	a
‘Seolhyang’	Con.	30.6	a	14.41	d	0.91	d	2.61	b	9	a	18.5	c	26.94	d	1.62	c	2	c
	W	27.5	b	17.38	c	1.23	c	2.84	b	11	a	22.3	b	34.64	b	1.69	c	3	b
	R	27.3	b	16.55	c	1.18	c	2.78	b	10	a	23.6	ab	32.85	c	1.52	c	3	b
	BR	25.9	b	20.25	b	1.52	b	3.29	a	12	a	24.5	a	35.90	b	2.04	b	4	a
	B	26.8	b	21.22	a	1.69	a	3.31	a	11	a	24.8	a	38.70	a	2.21	a	4	a
